# Behandlung der adulten chronisch nicht-bakteriellen Osteitis in Deutschland: Vergleich zwischen Behandlungsrealität und aktuellen Experten-Konsensempfehlungen

**DOI:** 10.1007/s00393-025-01766-1

**Published:** 2025-12-18

**Authors:** Pascal van Wijnen, Philipp Klemm, Michael Schmidt, Konstantinos Christofyllakis, Ulf Müller-Ladner, Gunter Assmann

**Affiliations:** 1 Abteilung für Rheumatologie und klinische, Justus-Liebig-Universität Giessen, ImmunologieCampus Kerckhoff, Bad Nauheim, Hessen Deutschland; 2 RUB-Universitätsklinikum Minden JWK, Klinik für Rheumatologie und klinische Immunologie, Ruhr University Bochum, Minden, Nordrhein-Westfalen Deutschland; 3 Jose Carreras Institut für Immun- und Gentherapie, Universitätsklinikum des Saarlandes, Homburg, Saarland Deutschland

**Keywords:** Krankheitsmodifizierende Antirheumatika, Real-World-Evidenz, Axiale Spondyloarthritis, Psoriasisarthritis, Klinische Überlappungen, Disease-modifying antirheumatic drugs, Real-world evidence, Axial spondyloarthritis, Psoriasis-arthritis, Clinical overlap

## Abstract

Die nicht-interventionelle Beobachtungsstudie analysierte mit einem retro- und prospektiven Design die Behandlungsrealität von 114 Patienten mit adulter chronisch nicht-bakterieller Osteitis (CNO) an drei deutschen universitären Zentren über den Zeitraum 1985 bis 2025. Primäres Ziel war der Abgleich der Therapieverläufe in Deutschland mit den im Jahr 2024 publizierten internationalen Konsensempfehlungen. Die Empfehlungen favorisieren nichtsteroidale Antirheumatika (NSAR) und Coxibe in der ersten und Tumornekrosefaktor-Inhibitoren (TNFi) oder Bisphosphonate in der zweiten Linie der Therapie.

Die Ergebnisse zeigen, dass zwar 61 % zuerst NSAR erhielten, aber nur 20 % in der empfohlenen Ausdosierung der Erstlinientherapie. Hingegen erhielten 46 % der adulten CNO-Patienten in der Erstlinie eine konventionell-synthetische Basistherapie (csDMARDs). Diese wird aufgrund der reduzierten Wirksamkeit aktuell nur in Einzelfällen empfohlen, wenn eine Überlappung der adulten CNO mit axialer Spondylarthritis (axSpA) oder Psoriasisarthritis (PsA) vorliegt. Unsere Daten zeigen, dass eine Überlappung in insgesamt 70 % der adulten CNO-Fälle auftrat. In der zweiten Linie der Therapie kamen wie empfohlen TNFi zum Einsatz, während die alternativ hierzu empfohlenen Bisphosphonate nur bei einem Patienten eingesetzt wurden.

Das beobachtete Therapieansprechen betrug, gemessen an der Rate der Therapieabbrüche, innerhalb von 12 Monaten maximal 53 %. Die vorliegenden Daten unterstreichen die Relevanz der aktuellen Empfehlungen zur Vereinheitlichung der Terminologie und zur Optimierung der therapeutischen Versorgung von Patienten mit adulter CNO. Ungelöst bleiben dabei die fehlende ICD-10-Codierung und Zulassung der durch die Konsensempfehlungen vorgeschlagenen Therapie für den deutschen Raum.

## Abstract (en)

The non-interventional observational study employed both retrospective and prospective approaches to analyze the treatment reality of 114 patients with adult chronic non-bacterial osteitis (CNO) at three German university centers over the period 1985–2025. The primary objective was to characterize treatment courses among adult CNO patients in Germany for and to compare these with the international consensus recommendations published in 2024. According to these recommendations, nonsteroidal anti-inflammatory drugs (NSAIDs) are preferred as first-line therapies, whereas tumor necrosis factor inhibitors (TNFi) or bisphosphonates are advised as second-line options.

The results indicate that although 61% of patients initially received NSAIDs, only 20% were treated at the recommended first-line dosage. In contrast, 46% of patients received conventional synthetic disease-modifying antirheumatic drugs (csDMARDs) as first-line therapy. Owing to their limited efficacy, csDMARDs are currently recommended only in selected cases with overlapping features of adult CNO and either axial spondyloarthritis (axSpA) or psoriatic arthritis (PsA). Our data demonstrate that overlap was present in 70% of the adult CNO cohort. As recommended, TNFi were the predominant second-line therapy (*n* = 32/86; 37%), whereas bisphosphonates were administered to only one patient.

The observed therapeutic response, assessed by the rate of treatment discontinuation within 12 months, reached a maximum of 53%. These findings underscore the importance of current consensus recommendations in standardizing terminology and optimizing treatment strategies for CNO. However, the lack of a dedicated ICD-10 code and the absence of regulatory approval for the recommended therapeutic agents in Germany remain unresolved challenges.

Sterile Knochenentzündungen („sterile bone inflammations“, SBI) wurden lange Zeit verschiedenen Krankheitsbildern wie der chronisch rekurrierenden multifokalen Osteomyelitis (CRMO), der diffus sklerosierenden Osteomyelitis (DSO), der sternokostoklavikulären Hyperostose (SCCH) und dem SAPHO-Syndrom (Synovitis, Akne, Pustulose, Hyperostose, Osteitis) zugeordnet. Obgleich häufig nicht alle Manifestationen des SAPHO-Syndroms zutrafen, wurden diese Fälle oftmals als unvollständige Ausprägung des Syndroms angesehen [1].

Erschwert wurde die Diagnosestellung und korrekte Einteilung durch die Heterogenität der Ausprägung der einzelnen Symptome, die Überlappung mit anderen rheumatologischen Krankheitsbildern und fehlender validierter Diagnose- und Klassifizierungskriterien [2]. Die Diagnose der adulten CNO basiert auf klinischen Manifestationen wie steriler Knochenentzündung, zu einem Teil begleitet von peripherer Arthritis und entzündlichen Dermatosen (wie Psoriasis oder Akne) sowie dem Ausschluss von Differenzialdiagnosen (Malignität, bakterielle Infektionen) [1]. Neben den Klassifizierungskriterien von Benhamou et al. von 1988, den Erstbeschreibern des SAPHO-Syndroms, sind die Kriterien von Kahn et al. aus dem Jahr 1994 am weitesten in der Fachwelt verbreitet [3–5]. Im November 2024 publizierte ein internationales Expertengremium die Empfehlung, zukünftig sämtliche Patienten mit SBI als „adulte chronisch nicht-bakterielle Osteitis“ (CNO) zu bezeichnen [6]. Es wurden klare Empfehlungen zur Definition, Diagnose und Behandlung der adulten CNO ausgesprochen. Hierdurch kam es erstmalig im Rahmen eines internationalen Expertenkonsens zu einer systematischen Vereinheitlichung der Nomenklatur und zu konkreten Handlungsempfehlungen für Patienten mit SBI [6]. Insbesondere sollten Bezeichnungen wie SAPHO, CRMO, DSO oder SCCH für Patienten mit adulter CNO nicht mehr als Primärbezeichnung verwendet werden.

Die Therapieempfehlungen wurden im Rahmen eines strukturierten Konsensusprozesses entwickelt. Mangels randomisierter Studien basiert die Evidenz damit überwiegend auf Expertenmeinungen, Fallserien und retrospektiven Daten. Zusammengefasst ist die CNO dabei in der Erstlinientherapie mit NSAR bzw. Cyclooxygenase-2-Inhibitoren (COX-II-Inhibitoren) zu behandeln [6]. In der Zweitlinientherapie sind intravenöse Bisphosphonate (BP) oder Tumornekrosefaktor-Inhibitoren (TNFi) anzuwenden. Bei unzureichendem Ansprechen kann in der Drittlinientherapie eine Kombination aus BP und TNFi erwogen werden, alternativ der Einsatz von anderen bDMARDs („biological disease-modifying antirheumatic drugs“) wie IL-17- oder IL-23-Inhibitoren. Die Wahl erfolgt dann individualisiert je nach klinischem Erscheinungsbild möglichst in einem für die Behandlung der CNO spezialisierten Zentrum [6].

Dies gibt Anlass, die deutsche Behandlungsrealität adulter CNO-Patienten zu untersuchen. In der folgenden Studie werden erstmals Therapieverläufe für Patienten in Deutschland aus den Jahren zwischen 1985 und 2025 dargestellt und anschließend mit den im Jahr 2024 publizierten Konsensempfehlungen abgeglichen. Weiterhin wurde untersucht, wie häufig eine Spondyloarthritis diagnostiziert wurde und ob dies einen Einfluss auf die Therapie hatte.

## Methodik

Die vorliegende Arbeit hat sowohl ein retrospektives als auch prospektives Design. Die Patientendatenbanken der universitären Zentren Homburg/Saar, Minden und Bad Nauheim/Gießen wurden nach Patienten durchsucht, welche im Zeitraum 1985–2025 wegen einer sterilen Knochenentzündung behandelt wurden. Die Patienten wurden in die retrospektive Auswertung eingeschlossen, wenn gemäß Aktenlage in ärztlicher Durchsicht eine sterile Entzündung an einem oder mehreren Knochen gemäß der neuen Definition der adulten CNO in den Konsensempfehlungen vorlag [6]. Diese musste in klinischer Untersuchung und Bildgebung nachvollziehbar sein. Zudem erfolgte ab 2024 der prospektive Einschluss aller CNO-Patienten der Standorte in eine nicht-interventionelle Beobachtungsstudie. Alle prospektiv eingeschlossenen Patienten erlaubten eine retrospektive Aufarbeitung des bisherigen Krankheits- und Therapieverlaufes. Es erfolgte eine deskriptive Erhebung der Patientencharakteristika (u. a. Alter, Geschlecht und humanes Leukozytenantigen [HLA]B27-Status), der extraossären Krankheitsmanifestationen (Psoriasis vulgaris, Psoriasis palmoplantaris pustulosa [PPP], periphere Arthritis, Sakroiliitis, chronisch-entzündliche Darmerkrankung oder schwere Akne) sowie des bisherigen Krankheitsverlaufes einschließlich Erkrankungsdauer. Schließlich wurden die aktuelle bzw. frühere Therapien inkl. Basistherapien (DMARD) ermittelt. Bei fehlenden validierten Messinstrumenten der Krankheitsaktivität einer adulten CNO wurde ein Therapieansprechen als eine Beibehaltung des gleichen Wirkstoffs in unveränderter Dosierung über einen Zeitraum von ≥ 12 Monaten definiert. Dabei wurde allen behandelnden Rheumatologen unterstellt, dass ein klinisches fehlendes Ansprechen zu einer Therapieumstellung führt analog des gängigen Treat-to-target-Prinzips in anderen Erkrankungen, z. B. axialer Spondylarthritis (axSpA) oder Psoriasisarthritis (PsA).

Darüber hinaus wurde untersucht, ob die CNO-Patienten Überlappungen zu Spondyloarthritiden aufwiesen. Hierfür wurde geprüft, ob die Klassifikationskriterien für eine axSpA oder PsA analog der Kriterien der ASAS (Assessment of SpondyloArthritis international Society) bzw. CASPAR (Classification Criteria for Psoriatic Arthritis) retrospektiv erfüllt gewesen wären bzw. ob eine solche Diagnose (im Overlap) im jeweiligen Fall tatsächlich gestellt wurde [7, 8].

Für die statistische Auswertung wurden die Programme R (Version 4.5.1) und Microsoft Excel verwendet. Zur Analyse kategorialer Daten kam der Pearson-Chi-Quadrat-Test und Fisher’s Exact-Test zum Einsatz. Ein Signifikanzniveau von *p* < 0,05 wurde als statistisch signifikant angesehen.

Die Untersuchung erfolgte entsprechend der Deklaration von Helsinki und wurde nach Genehmigung durch die erstbegutachtende Ethik-Kommission der Medizinischen Fakultät der Ruhr-Universität Bochum (Votum Nr. 2024-1273) und den entsprechenden Zweitbegutachtungen begonnen.

## Ergebnisse

### Patientencharakteristika

Es wurden 114 Patienten mit adulter CNO eingeschlossen und analysiert. Die meisten Patienten waren weiblich (*n* = 86; 75 %) mit einem mittleren Alter von 46 Jahren (SD = 13,34). Bei 23 Patienten (20 %) lag das Vollbild eines SAPHO-Syndroms vor (Tab. 1).

**Tab. 1:** Patientencharakteristika der adulten chronisch nicht-bakteriellen Osteitis (CNO)

	*N*	(%)
CNO	114	–
Weiblich	86	(75)
Durchschnittsalter bei Erstdiagnose (SD)	46	(13,34)
HLA-B27-positiv (*n* = 88)	14	(16)
Pustulose	66	(58)
Psoriasis vulgaris	43	(38)
Akne	30	(26)
Hidradenitis suppurativa	8	(7)
Sternokostoklavikuläre Osteitis (+/-Arthritis)	102	(89)
Sakroiliitis	36	(32)
Periphere Arthritis	43	(38)
SAPHO-Syndrom	23	(20)
CNO-Overlap	80	(70)
axSpA-Overlap*	35	(31)
PsA-Overlap*	31	(27)
axSpA- und PsA-Overlap*	14	(12)
ASAS- axSpA-Kriterien erfüllt	34	(30)
CASPAR-PsA-Kriterien erfüllt	39	(34)
ASAS + CASPAR-Kriterien erfüllt	12	(11)

*CNO* Chronisch nicht-bakterielle Osteitis, *SD* Standardabweichung, *HLA* Humanes Leukozytenantigen, *SAPHO* Synovitis, Akne, Pustulose, Hyperostose, Osteitis, *axSpA* Axiale Spondyloarthritis, *PsA* Psoriasisarthritis, *ASAS* Assessment of SpondyloArthritis international Society, *CASPAR* Classification Criteria for Psoriatic Arthritis

^*^gemäß Hauptdiagnose in den Arztberichten

Eine sternokostoklavikuläre Osteitis konnte bei 102 Patienten (89 %) nachgewiesen werden, bei den restlichen 12 Patienten waren u. a. Rippen, Femur, Os ilium oder Mandibula von SBI betroffen. Bildgebend (Magnetresonanztomographie oder Röntgenaufnahme) zeigte sich bei 36 Patienten (32 %) eine Sakroiliitis. Eine periphere Arthritis (exklusive der sternokostoklavikulären Arthritis) lag bei 43 Patienten (38 %) vor.

Begleitende dermatologische Manifestationen waren mit folgender Verteilung vorliegend: Eine PPP wurde bei 66 Patienten (58 %) dokumentiert, während 43 Patienten (38 %) eine Psoriasis vulgaris aufwiesen. Darüber hinaus litten 30 Patienten (26 %) an Akne; bei 8 dieser Patienten (7 %) handelte es sich um die schwere Subform der Hidradenitis suppurativa.

Der HLA-B27-Status war bei 88 Patienten bestimmt worden; davon waren 14 (16 %) Träger des HLA-B27-Gens.

### Therapiesequenz

#### Erstlinientherapie

Nach Aktenlage gaben 70 Patienten (61 %) bereits vor der rheumatologischen Erstvorstellung im jeweiligen rheumatologischen Zentrum an, bedarfsweise NSAR eingenommen zu haben, für eine Dauer zwischen 4 und 60 Monaten. Selten (< 10 %) wurden aber die NSAR rheumatologisch ausdosiert, wie in den Konsensempfehlungen besprochen und in Anlehnung an die medikamentöse Primärtherapie bei axSpA. Nach Diagnosestellung wurden 23 Patienten (20 %) im Rahmen der Erstlinientherapie mit NSAR in der empfohlenen rheumatologischen Dosierung für mindestens 3 Monate behandelt.

Der Großteil der Patienten erhielt eine nicht-NSAR-basierte Erstlinientherapie (Tab. 2).

**Tab. 2:** Erstlinientherapie bei adulter chronisch nicht-bakterieller Osteitis (*n* = 114).

	*n*	(%)	Dauer (Monate)	SD	Dauer ≥ 12 Monate (*n*)	(%)
*NSAR-Mono*	*23*	*(20)*	*28,1*	*48,87*	*12*	*(52)*
*csDMARD*	*53*	*(46)*	*27,5*	*49,62*	*22*	*(42)*
MTX	35	(66)	31,9	58,36	16	(46)
SSZ	15	(28)	18,6	27,22	4	(27)
HCQ	2	(4)	30,0	25,45	2	(100)
CYA	1	(2)	4,0	0,00	0	(0)
*bDMARD*	*28*	*(25)*	*23,6*	*27,64*	*14*	*(50)*
*TNFi*	*21*	*(75)*	*26,8*	*30,90*	*11*	*(52)*
ETN	9	(43)	31,1	39,33	5	(56)
ADA	7	(33)	23,7	15,35	5	(71)
IFX	3	(14)	30,0	45,90	1	(33)
CER	2	(10)	4,0	0,00	0	(0)
*IL-(12)/23i*	*2*	*(7)*	*7,5*	*4,90*	*0*	*(0)*
UST	1	(50)	4,0	0,00	0	(0)
GUS	1	(50)	11,0	0,00	0	(0)
*IL-17i*	*5*	*(18)*	*16,5*	*3,00*	*3*	*(60)*
SEC	3	(60)	21,0	3,00	3	(100)
BKZ	2	(40)	3,0	0,00	0	(0)
*tsDMARD*	*4*	*(4)*	*19,7*	*28,01*	*1*	*(25)*
*JAKi*	*3*	*(75)*	*27,5*	*34,50*	*1*	*(33)*
TOF	2	(67)	3,0	0,00	0	(0)
UPA	1	(33)	52,0	0,00	1	(100)
APR	1	(25)	4,0	0,00	0	(0)
*BP*	*1*	*(1)*	*6,0*	*0,00*	*0*	*(0)*
*Andere*	*5*	*(4)*	*10,6*	*9,21*	*2*	*(40)*
PRED-Mono	2	(40)	6,0	0,00	0	(0)
MES	2	(40)	19,0	7,00	2	(100)
Acitretin	1	(20)	3,0	0,00	0	(0)

*SD* Standardabweichung, *NSAR* Nichtsteroidales Antirheumatikum, *csDMARD* Konventionelles synthetisches Disease-Modifying Antirheumatic Drug, *MTX* Methotrexat, *SSZ* Sulfasalazin, *HCQ* Hydroxychloroquin, *CYA* Cyclosporin A, *bDMARD* Biologisches Disease-Modifying Antirheumatic Drug, *TNFi* Tumornekrosefaktor-Inhibitor, *ETN* Etanercept, *ADA* Adalimumab, *IFX* Infliximab, *CER* Certolizumab Pegol, *IL-(12)/23i* Interleukin-(12)/23-Inhibitor, *UST* Ustekinumab, *GUS* Guselkumab, *IL-17i* Interleukin-17-Inhibitor, *SEC* Secukinumab, *BKZ* Bimekizumab, *tsDMARD* Zielgerichtetes synthetisches Disease-Modifying Antirheumatic Drug, *JAKi* Januskinase-Inhibitor, *TOF* Tofacitinib, *BAR* Baricitinib, *UPA* Upadacitinib, *FIL* Filgotinib, *APR* Apremilast, *BP* Bisphosphonat, *PRED* Prednisolon, *MES* Mesalazin

csDMARDs wurden bei 53 Patienten (46 %) eingesetzt. Davon erhielten 35 Patienten (31 %) Methotrexat (MTX), 15 (13 %) Sulfasalazin (SSZ), 2 Patienten (2 %) Hydroxychloroquin (HCQ) und einer (1 %) Ciclosporin A (CYA).

Biologische DMARDs (bDMARDs) wurden bei 28 Patienten (25 %) angewendet. TNFi wurden bei 21 Patienten (18 %), IL-12/23-Inhibitoren bei 2 (2 %) und IL-17-Inhibitoren bei 5 der Patienten (4 %) eingesetzt.

Zusätzlich wurden 4 Patienten (4 %) mit zielgerichteten synthetischen DMARDs (tsDMARDs) behandelt. Drei Patienten (3 %) erhielten JAKi und ein Patient (1 %) Apremilast. Ein Patient (1 %) erhielt ein Bisphosphonat.

Weitere eingesetzte Substanzen waren Prednisolon als Monotherapie (*n* = 2; 2 %), Mesalazin (*n* = 2; 2 %) oder Acitretin (*n* = 1; 1 %).

Etwa 50 % der Patienten nahmen die Erstlinientherapie mit NSAR oder bDMARD über einen Zeitraum ≥ 12 Monaten ein, welches vorab als Therapieansprechen definiert worden war. Patienten unter csDMARDs, tsDMARDs und BP zeigten eine geringe Rate an Therapieansprechen (*n* = 22/53; 42 %; *n* = 1/4; 25 % bzw. *n* = 0/1; 0 %).

#### Zweitlinientherapie

Ein Großteil der analysierten Patienten (*n* = 86; 75 %) benötigte aufgrund unzureichender Wirksamkeit oder unerwünschter Arzneimittelwirkungen der Erstlinientherapie über den beobachteten Krankheitsverlauf eine zweite Therapie bzw. erhielt einen Therapiewechsel.

In der Zweitlinientherapie erhielten 32 der 86 Patienten (37 %) ein csDMARD. In der Erstlinie hatten davon 9 Patienten ein NSAR, 19 bereits ein anderes csDMARD, zwei ein bDMARD und zwei eine Monotherapie mit Prednisolon erhalten.

bDMARDs wurden bei 50 Patienten (58 %) eingesetzt. 32 Patienten (37 %) erhielten TNFi, 4 Patienten (5 %) bekamen IL-12/23-Inhibitoren, 12 Patienten (14 %) erhielten IL-17-Inhibitoren und jeweils ein Patient (1 %) Abatacept bzw. ein Patient (1 %) Tocilizumab.

In der Erstlinie waren diese Patienten zuvor mit NSAR (*n* = 7), csDMARD (*n* = 20), anderen bDMARD (*n* = 16), tsDMARD (*n* = 2) und BP (*n* = 1) behandelt worden.

**Abb. 1:**
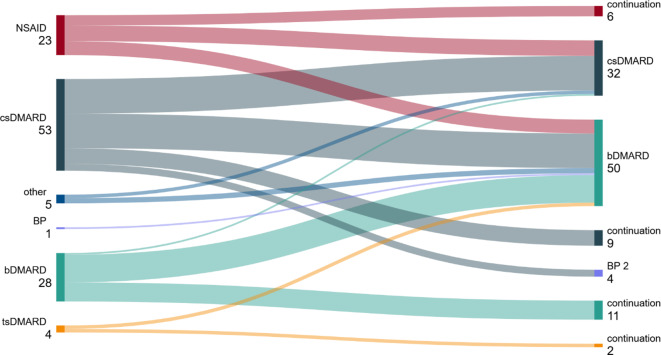
Sequenztherapie der adulten chronisch nicht-bakteriellen Osteitis (CNO) von Erst- auf Zweitlinie (*n* = 114). *NSAID* Nichtsteroidales Antirheumatikum, *csDMARD* Konventionelles synthetisches Disease-Modifying Antirheumatic Drug, *BP* Bisphosphonat, *bDMARD* Biologisches Disease-Modifying Antirheumatic Drug, *tsDMARD* Zielgerichtetes synthetisches Disease-Modifying Antirheumatic Drug, *other* andere Substanzen (Mesalazin, Prednisolon, Acitretin)

Eine Zweitlinientherapie mit BP wurde bei 4 Patienten (5 %) durchgeführt. Alle 4 Patienten hatten zuvor csDMARD in der Erstlinie erhalten (Abb. 1).

#### Drittlinientherapie

Bei 60 der 114 Patienten (53 %) war über den analysierten Beobachtungszeitraum eine Drittlinientherapie erforderlich. Dabei kamen erneut csDMARDs zum Einsatz (*n* = 7; 12 %).

Von den 60 adulten CNO-Patienten erhielten 43 (72 %) bDMARDs: In 27 Fällen (45 %) wurden TNFi verabreicht, in 7 Fällen (12 %) IL-12/23-Inhibitoren und in 9 Fällen (15 %) IL-17-Inhibitoren. Die Patienten hatten zuvor in der Zweitlinie csDMARD (*n* = 18), andere bDMARD (*n* = 23) und BP (*n* = 2) erhalten.

Darüber hinaus wurden 7 Patienten (16 %) mit dem tsDMARD Upadacitinib behandelt; andere tsDMARDs kamen in der Drittlinientherapie nicht zur Anwendung. Alle 7 Patienten hatten zuvor bDMARD in der Zweitlinie erhalten.

**Abb. 2:**
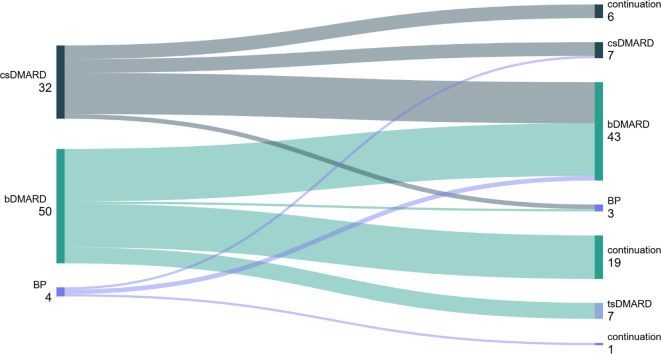
Sequenztherapie der adulten chronisch nicht-bakteriellen Osteitis (CNO) von Zweit- auf Drittlinie (*n* = 86). *csDMARD* Konventionelles synthetisches Disease-Modifying Antirheumatic Drug, *BP* Bisphosphonat, *bDMARD* Biologisches Disease-Modifying Antirheumatic Drug, *tsDMARD* Zielgerichtetes synthetisches Disease-Modifying Antirheumatic Drug

BP wurden bei 3 Patienten (5 %) angewendet. Zwei Patienten waren zuvor in der Zweitlinie mit csDMARD und ein Patient mit bDMARD behandelt worden (Abb. 2).

### Overlap axSpA/PsA

In den Arztberichten fand sich bei 80 Patienten (70 %) gleichzeitig eine Diagnose aus dem Formenkreis der Spondyloarthritiden. 35 Patienten der 114 Patienten (31 %) führten die Diagnose einer axialen Spondyloarthritis (axSpA), 31 Patienten der Kohorte (27 %) hatten eine (zusätzliche) Psoriasisarthritis (PsA) und bei 14 Patienten der Kohorte (12 %) bestand zusätzlich zur CNO sowohl die Diagnose einer PsA als auch axSpA.

Um die Überlappungen zwischen CNO und axSpA oder PsA weiter zu untersuchen, wurde die Erfüllung der ASAS- wie auch CASPAR-Kriterien für jeden Patienten anhand der vorliegenden Dokumentation retrospektiv analysiert und in einem zweiten Schritt mit der gestellten Diagnose verglichen.

Insgesamt erfüllten 34 von 114 Patienten (30 %) die ASAS-Kriterien der axSpA. 39 von 114 Patienten (34 %) konnten gemäß CASPAR‑Kriterien als PsA klassifiziert werden. Bei 12 Patienten (11 %) wurden sowohl die ASAS-Kriterien für eine axSpA als auch die CASPAR-Kriterien für eine PsA erfüllt.

Von den 35 Patienten mit CNO-axSpA-Overlap im Arztbrief wurden die ASAS-Kriterien jedoch nur in 60 % der Fälle erfüllt (Tab. 3). Auch die CASPAR-Kriterien wurden lediglich bei 17 von 31 diagnostizierten CNO-PsA-Patienten (55 %) erfüllt. Bei einem Overlap aus CNO-axSpA-PsA wurden nur bei 3 von 14 diagnostizierten Patienten (21 %) die ASAS- und CASPAR-Kriterien erfüllt.

**Tab. 3:** Vergleich der Diagnosen im Arztbrief mit der Erfüllung der Kriterien nach ASAS bzw. CASPAR-Klassifikation

	Arztbrief	ASAS/CASPAR	(%)
axSpA ohne PsA	35	21	(60)
PsA ohne axSpA	31	17	(55)
axSpA und PsA	14	3	(21)

*axSpA* Axiale Spondyloarthritis, *PsA* Psoriasisarthritis, *ASAS* Assessment of Spondyloarthritis international Society, *CASPAR* Classification Criteria for Psoriatic Arthritis

In einem letzten Schritt wurde analysiert, ob die (zeitgleiche) Diagnose einer axSpA oder PsA bei CNO Einfluss auf die Therapiesequenz hatte (Tab. 4).

**Tab. 4:** Angewendete Substanzklassen bei CNO-Patienten mit Overlap zu axSpA und/oder PsA.

	axSpA (*n* = 35)	(%)	PsA (*n* = 31)	(%)	axSpA und PsA (*n* = 14)	(%)	Ohne axSpA ohne PsA (*n* = 34)	(%)
NSAR	10	(29)	4	(13)	2	(14)	7	(21)
csDMARD	8	(23)	19	(61)	7	(50)	19	(56)
bDMARD	15	(43)	5	(16)	2	(14)	6	(18)
TNFi	12	(80)	4	(80)	0	–	5	(83)
IL12/23i	1	(7)	1	(20)	0	–	0	–
IL17i	2	(13)	0	(0)	0	–	1	(17)
tsDMARD	2	(6)	1	(3)	1	(7)	0	–
JAKi	2	(100)	0	–	1	(100)	0	–
PDE4i	0	–	1	(100)	0	–	0	–
BP	0	–	1	(3)	0	–	0	–
Andere	0	–	1	(3)	2	(14)	2	(6)

*CNO *chronisch nicht-bakterielle Osteitis,* axSpA* axiale Spondyloarthritis, *PsA* Psoriasisarthritis, *NSAR* Nichtsteroidales Antirheumatikum, *csDMARD* Konventionelles synthetisches Disease-Modifying Antirheumatic Drug, *bDMARD* Biologisches Disease-Modifying Antirheumatic Drug, *TNFi* Tumornekrosefaktor-Inhibitor, IL-(12)/23i Interleukin-(12)/23-Inhibitor, *IL-17i* Interleukin-17-Inhibitor, *tsDMARD* Zielgerichtetes synthetisches Disease-Modifying Antirheumatic Drug, *JAKi* Januskinase-Inhibitor, *PDE4i* Phosphodiesterase 4‑Inhibitor, *BP* Bisphosphonat

Patienten mit CNO und begleitender axSpA (*n* = 35) erhielten in der Erstlinie NSAR (*n* = 10; 29 %), csDMARD (*n* = 8; 23 %), bDMARD (*n* = 15; 43 %) oder tsDMARD (*n* = 2; 6 %).

Ein Overlap-Syndrom aus CNO und PsA (*n* = 31) wurde in der Erstlinie mit NSAR (*n* = 4; 13 %), csDMARD (*n* = 19; 61 %), bDMARD (*n* = 5; 16 %), tsDMARD (*n* = 1; 3 %), BP (*n* = 1; 3 %) sowie mit Acitretin (*n* = 1; 3 %) behandelt.

Bei Patienten mit CNO, axSpA und PsA wurden NSAR (*n* = 2; 14 %), csDMARD (*n* = 7; 50 %), bDMARD (*n* = 2; 14 %) und tsDMARD (*n* = 1; 7 %) und in zwei Fällen (14 %) Mesalazin eingesetzt.

Somit wurden Patienten mit CNO und axSpA häufiger mit bDMARD in der Erstlinie behandelt als Patienten mit CNO und PsA, jedoch war dieser Unterschied nicht signifikant (*p* = 0,1534). csDMARD wurden hingegen signifikant häufiger bei PsA eingesetzt (*p* = 0,0146) (Tab. 5).

**Tab. 5:** Vergleich der Therapieauswahl zwischen CNO-axSpA- und CNO-PsA-Overlap

	Nur axSpA (*n* = 35)	Nur PsA (*n* = 31)	*p*-Wert
NSAR	10	4	0,5713
*csDMARD*	*8*	*19*	*0,0146*
bDMARD	15	5	0,1534
tsDMARD	2	1	1,0000
BP	0	1	1,0000
Andere	0	1	1,0000

*CNO *chronisch nicht-bakterielle Osteitis,* axSpA* axiale Spondyloarthritis, *PsA* Psoriasisarthritis, *NSAR* Nichtsteroidales Antirheumatikum, *csDMARD* Konventionelles synthetisches Disease-Modifying Antirheumatic Drug, *bDMARD* Biologisches Disease-Modifying Antirheumatic Drug, *tsDMARD* Zielgerichtetes synthetisches Disease-Modifying Antirheumatic Drug, *BP* Bisphosphonat

## Diskussion

Die vorliegende Studie stellt mit 114 multizentrisch eingeschlossenen Patienten eines der bislang größten Kollektive von Patienten mit adulter CNO dar [9–11] und ist einzigartig im deutschsprachigen Raum.

Die führende Manifestation der adulten CNO ist die sternokostoklavikuläre Osteitis

Die führende Manifestation der adulten CNO ist die sternokostoklavikuläre Osteitis, welche in unserer Kohorte in 89 % der Fälle beobachtet wurde. Am zweithäufigsten bestanden kutane Manifestationen, allen voran die PPP, welche in 58 % der Fälle beobachtet wurde. Die Charakteristika der analysierten deutschen Patientenkohorte sind damit grundsätzlich vergleichbar mit weltweit publizierten CNO-Kollektiven. Die eine Häufigkeit der PPP zwischen 37 und 68 % der CNO-Patienten angeben, wobei hier auch Patienten mitgezählt werden, die zwar keine aktive PPP, aber eine positive Erkrankungshistorie hatten. In unserer Kohorte konnte bei 58 % der CNO-Patienten eine aktive PPP nachvollzogen werden.

Primäres Ziel der Studie war es, Therapieverläufe für Patienten mit adulter CNO in Deutschland darzustellen und anschließend mit den im Jahr 2024 publizierten Konsensempfehlungen einer internationalen Expertenkommission abzugleichen.

Diese empfiehlt nach Diagnosestellung der adulten CNO zunächst eine maximaldosierte NSAR-Therapie über 2–4 Wochen. Deutlich abweichend wurde eine solche Therapie nur bei 20 % der Patienten in der Erstlinie rheumatologisch begonnen. Einschränkend ist anzumerken, dass die Mehrheit der Patienten (61 %) bei Erstvorstellung berichtete, bereits im Vorfeld NSAR vergeblich (bedarfsweise) eingenommen zu haben. Limitierend kommt hier das Studiendesign zum Tragen: Bei den retrospektiv erhobenen Krankheitsverläufen kann die NSAR-Behandlung nur anhand der dokumentierten Anamnese nachvollzogen werden. In den prospektiv erhobenen Patientenfällen wird die Vorbehandlung bei Erstvorstellung umfänglicher (z. B. Dosierung, Effekte, Zeitraum) erhoben.

Im Gegensatz zu den Konsensempfehlungen wurde fast die Hälfte der Patienten (46 %) in der Erstlinie mit csDMARD behandelt. Darunter kam es bei 42 % der Patienten zu einem Therapieansprechen, welches als Weiterverordnung des Wirkstoffs in unveränderter Dosierung über mindestens 12 Monate definiert wurde. Dies steht im Kontrast zu Beobachtungen in niederländischen Kollektiven, in welchen csDMARD nur bei 8 % der CNO-Patienten erfolgreich waren [10]. Eine mögliche Erklärung hierfür kann der deutliche Anteil von aktiver PPP (58 %) in unserem CNO-Kollektiv sein, wodurch sich unsere Kohorte von den weltweit publizierten Kohorten mit adulter CNO möglicherweise unterscheidet. Die PPP ist eine oft therapierefraktäre und belastende Krankheitsausprägung, für die eine paradoxe Reaktion auf TNFi in Einzelfällen beschrieben wurde und welche die Therapieauswahl für die adulten CNO auch nach unserer Erfahrung damit durchaus beeinflusst haben kann [15]; eine Erklärung für das Übergewicht von csDMARDs, insbesondere mit MTX, HCQ und Ciclosporin gerade im Kollektiv von 1985–2010 vor der Ära der bDMARD jenseits der TNFi erscheint zumindest vor diesem Hintergrund plausibel.

Unsere Definition des Therapieansprechens als unveränderte Medikation über ≥ 12 Monate ist eine klare Limitation der Studie, da es sich nicht um einen präzisen Parameter handelt und die Gründe für die Fortsetzung bzw. den Abbruch der Therapie vielfältig sind. Einerseits kann bei der oben ausgeführten Definition nicht nachvollzogen werden, ob die Medikation wegen einer Besserung der Osteitis oder wegen einer Besserung sonstiger Symptome wie beispielsweise der extraossären Manifestationen wie PPP oder Akne weiterverordnet wurde. Andererseits reichen die Gründe für einen Therapieabbruch bei den Verlaufskontrollen von Unverträglichkeitsreaktionen über Wirkungslosigkeit bis hin zu Incompliance. Aufgrund des retrospektiven Studiendesigns für den überwiegenden Teil der untersuchten Kohorte und mit einem untersuchten Zeitraum von 40 Jahren sowie unterschiedlichen schwer vergleichbaren diagnostischen Methoden war eine solche Definition – mit allen genannten Einschränkungen – jedoch letztlich der am besten objektivierbare Parameter. Die Definition unterstellt den behandelnden Rheumatologen insgesamt ein Treat-to-target-Prinzip in ihrer Behandlung bzw. eine Umstellung bei fehlenden klinisches Ansprechen der Therapie – unabhängig von (den hier fehlenden) validierten Krankheitsaktivitätsparametern.

Eine weitere Limitation unserer Studie stellt die Heterogenität der Kohorte über den Zeitraum von 40 Jahren dar, in dem die in den Konsensempfehlungen vorgeschlagene Behandlungsindikation bei positiven klinischen und bildgebenden Befunden (vorzugweise mittels Magnetresonanztomographie [MRT]) nicht mehr nach heutigen Standards nachvollzogen werden konnte – sei es aufgrund der zum Teil technisch unzureichenden MRT-Bildqualität oder aufgrund alternativer bildgebender Verfahren wie der Computertomographie (CT) und/oder Skelettszintigraphie für den Zeitraum vor ca. 2010.

In der Erstlinientherapie bestanden signifikante Unterschiede zwischen adulten CNO-Patienten mit und ohne Overlap-Erkrankungen: Die Patienten mit zeitgleicher Diagnose einer PsA wurden signifikant häufiger mit csDMARDs behandelt als Patienten mit zeitgleicher Diagnose einer axSpA (61 % vs. 23 %). Hier erstaunt, dass selbst axSpA Patienten in 23 % der Fälle eine csDMARD-Therapie erhielten (Tab. 4). Basierend auf der nach Datenlage eher ungenügenden Wirksamkeit von csDMARD bei adulter CNO empfiehlt die Konsensus-Konferenz lediglich bei begleitender Polyarthritis ein csDMARD [6]. Eine periphere Arthritis lag jedoch nur bei 38 % der Patienten bei Erstdiagnose vor. Zudem fällt auf, dass in der Erstlinie bereits bei 25 % aller Patienten bDMARDs eingesetzt wurden. Bei Patienten mit einer adulten CNO und axSpA erhielten sogar 35 % eine bDMARD-Erstlinientherapie.

Insgesamt 86 Patienten (75 %) benötigten eine Therapieumstellung, d. h. Zweitlinientherapie. 37 % dieser Patienten erhielten csDMARD. Dabei wurden 23 % der Patienten der Zweitlinien-Kohorte sowohl in der Erst- als auch in der Zweitlinie mit csDMARD sequenziell behandelt. Der Anteil an Patienten mit einer bDMARD-Therapie erhöhte sich auf 58 %. Die aktuelle Konsensempfehlung zum bevorzugten Einsatz von BP als Zweitlinientherapie wurde lediglich bei insgesamt 4 von 86 Patienten (5 %) umgesetzt [6, 12]. Die alternativ zu BP empfohlenen TNFi wurden immerhin bei 32 CNO-Patienten (37 %) angewendet. Hiervon hatten 23 CNO-Patienten einen Overlap zu axSpA und/oder PsA, so dass diese Therapiewahl in zweierlei Hinsicht gut begründet scheint.

Über die Hälfte der Patienten benötigte eine Drittlinientherapie. Der Anteil von bDMARD war in der Drittlinie am höchsten (72 %), wovon wiederum die TNFi mit 45 % den größten Teil ausmachten. Bisphosphonate wurden in 5 % der Fälle als Monotherapie eingesetzt. Die dabei empfohlene Kombinationstherapie aus TNFi und BP wurde in unserem Kollektiv kein einziges Mal angewandt. Einschränkend muss in dem Zusammenhang die fehlende Zulassung von BP in der Therapie der adulten CNO in Deutschland erwähnt werden.

Im Vergleich zu den Konsensempfehlungen ist die Behandlungsrealität in Deutschland deutlich diverser und unser Kollektiv weist hier in der Retrospektive einzelne zum Teil deutliche Unterschiede zu den aktuellen Behandlungsempfehlungen auf. Gerade die empfohlene strikte Hierarchie aus NSAR → BP oder TNFi → BP und TNFi fand in unserem Kollektiv bezüglich des Einsatzes von BP kaum Anwendung. TNFi hingegen stellen bis zu 75 % der eingesetzten bDMARDs, bei Versagen kommen jedoch häufiger bDMARDs mit anderer Wirkweise oder tsDMARDs zum Einsatz.

Bei nur 30 % der adulten CNO-Patienten wurde keine axSpA oder PsA diagnostiziert. 31 % hatten eine zeitgleiche axSpA, 27 % eine PsA. Klinisch wiesen die Patienten oftmals Merkmale einer Spondyloarthritis auf, jedoch lassen sich Patienten mit adulter CNO meistens nicht einer klassischen Spondyloarthritis zuordnen. Die Anwendung etablierter Klassifikationskriterien (ASAS, CASPAR) zur Überprüfung der ärztlich gestellten Diagnosen zeigte, dass ein substanzieller Anteil nicht die (Klassifikations‑)Kriterien erfüllte. Die Diskrepanz zwischen den in den Arztbriefen formulierten Diagnosen und den nichterfüllten ASAS-/CASPAR-Klassifikationskriterien legt nahe, dass adulte CNO-Patienten möglicherweise gezielt unter diesen Diagnosen geführt werden. Eine plausible Erklärung dafür könnte die regulatorische Situation sein: Da in Deutschland für Patienten mit adulter CNO kein ICD-10-Code existiert und bislang keine spezifischen Arzneimittel zugelassen sind, greifen behandelnde Ärzte in einzelnen Fällen auf Diagnosen wie axSpA oder PsA zurück, um eine Abrechnung ihrer Leistungen sowie eine Therapie mit zugelassenen Immunmodulatoren zu ermöglichen. Diese Praxis würde einerseits sicherstellen, dass Patienten Zugang zu wirksamen Medikamenten erhalten, erschwert jedoch die retrospektive Auswertung dieses Kollektives hinsichtlich der Therapieindikationen. Zu diese Sichtweise passt auch, dass unser Kollektiv höhere Raten an Überlappungen von axSpA und PsA mit der adulten CNO aufweist, als in den Konsensempfehlungen angegeben. Dass die CNO zwar Gemeinsamkeiten mit dem Formenkreis der Spondyloarthritiden aufweist, jedoch als eigenständige Erkrankung aufgefasst werden muss, bleibt damit aber unbestritten. Unterstrichen wird diese Interpretation durch die erhobenen Therapieverläufe der adulten CNO in unserem Kollektiv, welche sich oftmals dem Schema der PsA oder axSpA bedienen, jedoch eine maximale Ansprechrate hinsichtlich der adulten CNO von nur 50 % erzielen. Im Vergleich zu spezifischen und krankheitsadaptierten Therapiealgorithmen wie bei der rheumatoiden Arthritis mit Behandlungserfolgen von bis zu 65 %, ist dies deutlich weniger [13–15].

## Fazit für die Praxis

- Bei der adulten chronisch nicht-bakteriellen Osteitis (CNO) handelt es sich um eine eigenständige Entität.
- In der deutschen Behandlungsrealität zeigte sich im Vergleich zur aktuell publizierten Konsensempfehlung ein uneinheitliches und von aktuellen Behandlungsempfehlungen divergierendes Vorgehen.
- Insbesondere der häufige Einsatz von konventionell-synthetischen krankheitsmodifizierenden Arzneimitteln (csDMARDs) und Bisphosphonaten (BP) fällt auf. Dies kann einerseits als bestehende Unsicherheit im klinischen Alltag gedeutet werden, kann aber auch durch das Fehlen von ICD-10-Codes für die adulte CNO sowie jeglicher Zulassung teurer Medikamente bedingt sein.
- Die diagnostische Zuordnung vieler Patienten zur axialen Spondyloarthritis (axSpA) oder Psoriasisarthritis (PsA) ist nicht nur auf klinische Überlappungen zurückzuführen, sondern auch auf den möglichen Zugang zu zugelassenen Therapien.
- Die aktuell publizierten Konsensempfehlungen zu einem stufenweisen Therapiekonzept scheinen somit nicht nur maßgeblich und sinnvoll, sondern auch für die Zukunft vielversprechend.

## Data Availability

Die erhobenen Datensätze können auf begründete Anfrage in anonymisierter Form beim korrespondierenden Autor angefordert werden.
